# Integrated Biorefinery of Rotted Date Fruits: One-Pot Co-Production of Lipids and Pigments by *Talaromyces atroroseus* PZ091940 and Valorization of Residual Biomass Wastes for Fungal Chitosan

**DOI:** 10.3390/biology15090688

**Published:** 2026-04-28

**Authors:** Diana A. H. Al-Quwaie

**Affiliations:** Biological Sciences Department, College of Science & Arts, King Abdulaziz University, Rabigh 21911, Saudi Arabia; dalquwaie@kau.edu.sa

**Keywords:** *Talaromyces atroroseus*, spoilage date fruits, biodiesel production, natural pigments, fungal chitosan

## Abstract

Spoilage date palm fruits are produced in large quantities and represent an underutilized agrowaste resource. Their high sugar content and balanced nutrient composition make them promising candidates for microbial bioprocessing. This study explored their potential as a low-cost substrate for *Talaromyces atroroseus* QA2602 (PZ091940) to simultaneously produce biodiesel grade lipids, natural pigments, and fungal chitosan within an integrated biorefinery approach. Spoiled date fruits were chemically characterized and applied at varying concentrations to cultivate *T. atroroseus* QA2602 (PZ091940). Thermal and thermo-chemical pretreatments were tested to enhance sugar availability. Lipid accumulation, fatty acid methyl esters (FAMEs) profiles, pigment production, and pigment stability were assessed. Biodiesel quality was estimated from FAME composition. De-oiled fungal biomass wastes were further processed to extract and characterize chitosan, and pigment–chitosan composites were evaluated for antioxidant activity. Optimal lipid and pigment production by *T*. *atroroseus* occurred at moderate concentration of spoiled date fruit substrate used in the culture medium, while dilute acid pretreatment of spoiled date fruits at high temperature resulted in the highest reducing sugar release from the substrate, which subsequently enhanced fungal biomass formation. The resulting C16–C18 rich oil displayed fuel properties consistent with high quality biodiesel. Pigments exhibited strong pH and thermal stability, along with potent antioxidant activity. De-oiled biomass produced chitosan with a high degree of deacetylation, and the pigment–chitosan composite showed enhanced antioxidant capacity. Rotted date fruits provide an effective, sustainable feedstock enabling the co-production of biodiesel, pigments, and chitosan by *Talaromyces atroroseus* QA2602 (PZ091940), supporting their integration into circular bioeconomy frameworks.

## Simple Summary

Large quantities of spoiled date palm fruits are generated annually and are often discarded, resulting in environmental and economic losses. Developing sustainable strategies to convert this waste into valuable products is therefore an important challenge. This study aimed to establish an integrated bioprocess that utilizes spoiled date fruits as a low-cost substrate to simultaneously produce biodiesel-grade lipids, natural pigments, and fungal chitosan using *Talaromyces atroroseus*. The objectives included optimizing substrate concentration and pretreatment conditions, evaluating lipid and pigment production, and recovering chitosan from residual fungal biomass. The results demonstrated that moderate substrate levels supported maximum lipid and pigment yields, while dilute acid pretreatment significantly enhanced sugar release from spoiled date fruits and fungal productivity. The extracted lipids were rich in C16–C18 fatty acids and complied with international biodiesel quality standards. The produced pigments showed good pH and thermal stability along with strong antioxidant activity. Additionally, chitosan recovered from de-oiled biomass exhibited suitable structural properties, and pigment–chitosan composites displayed enhanced antioxidant performance. Therefore, this work presents a sustainable biorefinery approach that transforms agricultural waste into multiple high-value products, supporting waste valorization, renewable energy production, and circular bioeconomy initiatives and offering clear benefits to society and industry.

## 1. Introduction

The international shift towards low-carbon energy systems has been accelerated in recent years by increasing oil prices, worsening effects of climate change, and rising energy security concerns. Governments worldwide have been improving their policy structures to support the use of renewable energy sources, decrease dependence on fossil fuels, and address environmental concerns [1,2]. In this regard, biofuels are considered a key component because they are biodegradable, have the potential to lower life-cycle greenhouse gas emissions, and can be produced on a large scale to meet the growing demands of the international community for energy. Among the different types of liquid biofuels, biodiesel is considered most promising because of its high energy density and non-toxic combustion properties, making it a potential alternative for the current environmental pollution and future energy shortages [3,4].

A promising route for biodiesel production involves the use of filamentous fungi as microbial factories. Many oleaginous fungal species are capable of accumulating large quantities of intracellular lipids—known as single-cell oils (SCOs)—that closely resemble the fatty-acid composition of vegetable oils used for biodiesel synthesis. Species such as *Mortierella alpina*, *Mucor circinelloides*, *Aspergillus oryzae* and *Penicillium citrinum* can accumulate more than 20–70% lipid in their dry biomass when grown under nitrogen-limited conditions [5,6,7,8]. Fungal systems offer additional advantages, including rapid growth rates, tolerance to variable substrate quality, and the ability to utilize low-cost feedstocks such as lignocellulosic hydrolysates, agro-industrial residues, and certain wastewater streams [9,10]. These properties reduce production costs and enhance sustainability. Moreover, fungal mycelia can be harvested easily through simple filtration, unlike microalgae that require energy-intensive dewatering. Continued progress in metabolic engineering and fermentation design is steadily improving SCO productivity and enriching desirable C16–C18 fatty acids, strengthening the potential of fungi as efficient biodiesel producers [11,12].

Parallel to advances in biofuels, the global market for natural pigments is expanding rapidly, with projections exceeding $33 billion by 2027 [13]. Microbial pigments represent one of the fastest-growing segments due to increasing consumer demand for natural, safe, and eco-friendly colorants [13]. Synthetic dyes, though still dominant because of their low cost, face increasing scrutiny due to documented health and environmental risks—including potential links between certain food dyes and behavioral effects in children [14,15]. Plant-based pigments, historically the primary natural alternatives, are limited by seasonality, batch variability, and high production costs [16]. Microbial pigments overcome these limitations by offering continuous production, controlled cultivation, and broad chemical diversity.

Filamentous fungi are particularly valuable sources of natural pigments, including azaphilones, anthraquinones, and naphthoquinones [17,18]. Among these, *Talaromyces* spp. is a known producer of red pigments whose yield and color properties can be optimized through pH, temperature, and nutrient composition [19]. The red-pigment-producing fungus *Monascus purpureus* has long been used in southern China, Japan, and southeast Asia for the production of red rice wine, fermented soybean products, and red yeast rice (Anka). However, its industrial application is limited by safety concerns, as some *Monascus*-fermented products contain the mycotoxin citrinin, and many strains also produce mevinolin, a pharmaceutical compound undesirable in foods. In contrast, citrinin has not been reported in *Talaromyces* species, suggesting that *Talaromyces* may represent a safer alternative for red pigment production [20]. Furthermore, pigment production in this species is not strictly tied to growth, enabling a two-phase fermentation strategy in which biomass is first accumulated under nutrient-balanced conditions, followed by induction of pigment synthesis through nitrogen limitation or specific pH ranges [19,21]. Although reports of SCO accumulation by *T*. *purpureogenus* are limited, its metabolic flexibility and ability to grow on agro residues suggest potential for integrated production of lipids and pigments when cultivation phases are carefully managed [18,22].

Bioprocess economics are strongly influenced by medium costs, which may account for up to 30% of total expenses [23]. Therefore, the valorization of agro-industrial residues as fermentation substrates is essential for large-scale feasibility. Date-processing residues, including date pomace and downgraded fruits, offer similar opportunities. These byproducts are abundant in fermentable sugars, pectin, phenolics, and organic acids. Mild pretreatment methods—including milling, hot-water extraction, or chemical hydrolysis—can produce sugar-rich media suitable for fungal growth and secondary metabolism [24,25]. Various studies have demonstrated the successful conversion of date waste into organic acids, ethanol, polymers, and other value-added products, confirming their potential as low-cost substrates [24,26]. This opportunity is highly relevant in Saudi Arabia, where date production exceeds million tonnes annually, and exports continue to rise across many global markets [27]. Such large-scale production generates significant amounts of underutilized residues, including downgraded fruits, syrup sludges, and seed-rich pomace. These residues are rich in fermentable sugars, lipids, fibers, and bioactive compounds suitable for microbial upgrading [25]. Saudi Arabia’s circular-economy initiatives—which emphasize waste reduction, recycling, and domestic bioindustry development—further support the integration of date waste into regional.

Within this context, the biorefinery concept offers clear advantages by generating multiple products from a single feedstock, improving carbon utilization, and spreading production costs across several product streams [25]. For rotted date fruits, an integrated fungal biorefinery could produce SCO-based biodiesel, natural fungal pigments, and fungal chitosan recovered from spent biomass. The extraction of chitosan is particularly valuable because fungal cell walls contain chitin–chitosan structures that can be recovered after lipid extraction. Chitosan can be produced from either crustacean shells or fungal biomass, representing two distinct supply routes with different advantages and limitations. Crustacean shells—primarily derived from shrimp, crab, and lobster waste—are the most established industrial source and remain dominant due to their relatively high chitin content (6.0–25.0%). However, this route is constrained by seasonal and limited raw material availability, intensive use of strong acids and alkalis, and high energy demands associated with harsh processing conditions (30–50% alkali and temperatures above 100 °C). Additional challenges include the need for extensive demineralization due to high calcium carbonate content and difficulty in producing highly deacetylated chitosan suitable for biomedical applications [28,29,30].

In contrast, fungal chitosan is obtained from the cell walls of filamentous fungi, where chitin exists as a chitin–glucan complex with generally lower chitin content (8–16%). Despite this, fungal sources offer several compelling advantages. Fungal chitosan can be produced year-round under controlled fermentation conditions, independent of marine resources and seasonal seafood processing. The absence of calcium carbonate eliminates harsh demineralization steps, reducing chemical use and wastewater generation. Moreover, fungal chitosan is free from shellfish allergens, enhancing its suitability for biomedical, pharmaceutical, and food applications. Its production can also be integrated into fermentation-based biorefineries as a co-product of lipid, enzyme, or pigment synthesis, improving sustainability and economic efficiency [28,29,30]. Based on this rationale, the present study aimed to develop an integrated fungal biorefinery that simultaneously produces biodiesel precursors (SCOs) and fungal pigments, followed by the recovery of high-quality fungal chitosan from the remaining biomass. By using rotted date byproducts as a low-cost substrate and applying staged cultivation with green downstream processing, the study seeks to maximize product yields while contributing to regional sustainability and circular-economy goals.

## 2. Materials and Methods

### 2.1. Substrate Preparation and Characterization

Low-grade spoiled date palm fruits that did not meet the human consumption or commercial sale standards were collected and washed twice with sterile distilled water. A 50 g portion of the cleaned fruits was macerated and homogenized with 200 mL of sterile distilled water in a blender for 10 min. The pulp thus formed was boiled for 10 min, cooled and filtered through two layers of muslin cloth. The filtrate was diluted with water to make a final volume of 1 L. The pH of the medium was adjusted to 6.8 using 2 M NaOH or 2 M HCl. The biochemical composition of the spoiled date fruits was determined by using the standard chemical methods. Protein content was measured by the Coomassie Brilliant Blue G 250 dye binding assay as described by Bradford [31] and using the bovine serum albumin (BSA) as standard. Total lipids were determined by colorimetric analysis using the Sulfo-phosphovanillin method [32]. Reducing sugars were determined colorimetrically by the dinitrosalicylic acid method [33], whereas total sugars were quantified by the anthrone-sulfuric acid method [34]. Free amino acids were determined following Muting and Kaiser [35] protocol. The ash obtained from rotten date fruit samples was used for mineral analysis. Sodium and potassium contents were determined by flame photometry while calcium and magnesium levels were measured by EDTA (Versene) titration [36]. Trace elements such as zinc, copper, iron, and cobalt were evaluated by atomic absorption spectrometry (210 VGP Buck Scientific, Ansonia, CT, USA). All measurements were done three times.

### 2.2. Microorganism and Inoculum Preparation

*Talaromyces* sp. isolate number QA2602 was cultivated on PDA plates and incubated for 6 days at 28 ± 2 °C. The growing fungal mycelia were carefully taken off the surface of the plates and blended with sterile distilled water to get a consistent spore suspension.

### 2.3. Identification of Talaromyces sp. QA2602

The identification of *Talaromyces* sp. QA2602 was performed through analysis of its 18S rRNA gene following the procedure described by Tawfik et al. [37]. The resulting 18S rRNA sequence was subjected to similarity searching using the advanced BLAST tool (BLAST+ 2.16.0) available on the NCBI website (http://www.ncbi.nlm.nih.gov/BLAST/, accessed on 1 January 2020) to determine its closest genetic matches. The nucleotide sequence of *Talaromyces atroroseus* QA2602 has been submitted to the GenBank database under the accession number PZ091940.

### 2.4. Culture Medium and Cultivation Condition

The fungus *Talaromyces atroroseus* QA2602 was grown in a liquid culture medium containing (g L^−1^): different substrate concentrations (25, 50, 75, 100, 150 and 200 g L^−1^ of spoiled date fruits); peptone, 2; and chloramphenicol, 0.25. The medium was sterilized by autoclaving and then 1 mL of spore suspension (1 × 10^6^ spores mL^−1^) was used to inoculate 50 mL of culture medium in 100 mL Erlenmeyer flasks. Flask cultures were incubated at 120 rpm for 10 days.

### 2.5. Biomass Dry Weight Determination

Fungal biomass was collected from the broth by centrifugation at 6000 rpm for 5 min to separate the extracellular fungal pigments in the supernatant from the fungal biomass pellet. The recovered mycelium was rinsed with distilled water three times to wash off the leftover medium components and was then dried at 65 °C. Later, the dried biomass was weighed, and the dry weight was recorded and kept for assay lipid content.

### 2.6. Lipid Content Assay

Lipid content was quantified by means of the Sulfo-phosphovanillin method [32]. The phosphovanillin stock reagent was made by dissolving 0.6 g of vanillin in 10 mL of absolute ethanol; after that the solution was diluted with deionized water to 90 mL, and finally 400 mL of concentrated phosphoric acid was added. For the Sulfo-phosphovanillin assay, 1 mg of dried fungal biomass was first resuspended in 1 mL of chloroform:methanol 2:1 *v*/*v*; then, 100 µL of the extract was mixed with 2 mL of concentrated sulfuric acid (98%). The mixture was first incubated at 100 °C for 10 min and then rapidly cooled down in an ice bath. Then, 1.5 mL of phosphovanillin reagent was added to 50 µL of the mixture, and the reaction mixture was incubated at 37 °C with shaking at 200 rpm for 15 min. After the incubation step, the absorbance of the supernatant obtained was determined at 530 nm and the lipid content was recorded. Lipid content was quantified by comparing the measured optical density at 530 nm with a standard calibration curve prepared using known concentrations of a lipid standard sunflower oil.

### 2.7. Pretreatment Methods of Spoilage Date Fruits for Enhancement of Lipid and Pigment Productivity

Spoilage date fruits were subjected to various pretreatment methods including thermal, chemical (acid and alkaline), and thermo-chemical to increase lipid and pigment production by *Talaromyces atroroseus* QA2602. Chemical pretreatment of the spoilage date fruits was done by treating the spoilage date fruits in liquids of different concentrations of sulfuric acid (0.05, 0.1 and 0.2 M) or sodium hydroxide (0.5, 1.0 and 1.5 M). The samples were shaken at 150 rpm for 30 min to make sure that the pretreatment solutions were uniformly exposed to the samples. Then, the containers were subjected to different thermal conditions for 20 min. Each treatment, including the control without treatment, was done in triplicate. After heat treatment, the pH of each sample was measured and corrected to 6.8 with either 0.1 N HCl or NaOH before fermentation, and the samples were incubated under shaking at 28 °C for 10 days. The total sugar content of the spoilage date fruits before and after fermentation was determined by the anthrone-sulfuric acid method [34]. The produced fungal lipid and pigment was estimated.

### 2.8. Biodiesel Production and Characterization from Fungal Lipids

In this study, biodiesel was prepared by firstly performing an acid catalyzed transesterification of the fungal lipids [38]. The obtained biodiesel layer containing the fatty acid methyl esters (FAMEs) was injected into the GC/MS (Agilent 6890N/5975B, Agilent Technologies, Inc., Santa Clara, CA, USA) for analysis. The amount of FAME obtained was estimated from the correspondences of the peak areas against the internal standards. The quality of the resulting biodiesel was assessed by calculating key physicochemical parameters using the following equations and comparing them with EU (EN 14214) and US (ASTM D6751) standards [39,40]. Density (ρ) was estimated from the mass fraction weighted densities of individual FAMEs; kinematic viscosity (ν_mix_) was calculated by summing the contributions of each ester based on its relative abundance. Saponification (SN) and iodine values (IVs) were determined using molecular weight and degree of unsaturation of each FAME, respectively. The higher heating value (HHV) was estimated from the saponification and iodine values, while the cetane number (CN) was calculated using the reported CN of each pure FAME weighted by its mass fraction.

$$Density\left(p\right)=\sum (c_{i}\;\times \;\rho_{i})$$

$$Kinematic\;viscosity\;(\nu_{mix})=\sum (A_{c}\times v_{c})$$

$$Saponification\;number\;(SN)=\sum (254\times A_{i})/MW_{i}$$

$$Iodine\;value\left(IV\right)=\sum (254\;\times \;D\times A_{i})/MW_{i}$$

$$Higher\;heating\;value\left(HHV\right)=49.43-[0.041\left(SN\right)+0.015\left(IV\right)]$$

$$Cetane\;number\left(CN\right)=1.068\sum \left(CN_{i}\;\times \;W_{i}\right)-6.747$$

C_i_ = the concentration (mass fraction) of each fatty acid methyl ester (FAME) present in the biodiesel;

ρ_i_ = the density of each individual FAME component in the biodiesel;

A_c_ = the relative abundance of each ester in the biodiesel sample, as determined by GC–MS analysis;

ν_c_ = the kinematic viscosity value of each FAME, obtained from standard viscosity reference data;

A_i_ = the percentage of each FAME measured by GC–MS;

MW_i_ = the molecular weight of each FAME;

D = the number of carbon–carbon double bonds in each FAME molecule;

CN_i_ = the reported cetane number of each pure FAME from published databases;

W_i_ = the mass fraction of each FAME quantified by GC–MS.

### 2.9. Assay and Characterization for Fungal Pigments

Extracellular pigments from the culture were obtained off the biomass by vacuum filtration. The filtrate was gathered in 50 mL sterile centrifuge tubes and kept in storage for later use. Fungal pigment analysis was done by two different methods as follows: the crude fungal pigment extracts were subjected to a full wavelength scan from 200 to 800 nm using a UV scanning spectrophotometer to identify the characteristic absorption peaks. The pigment concentration was determined by measuring absorbance at the isolate, specific maximum wavelength (obtained from the UV scan), and Vis spectrophotometer (L7 UV/Visible spectrophotometer, Taisite Lab (New York, NY, USA)). The un-inoculated broth medium served as the control. Pigment concentration was calculated according to the Beer–Lambert law:

A=a b c

where *A* = absorbance of the pigment, *a* = molar absorptivity constant, *b* = path length of the cuvette, and *c* = pigment concentration.

#### 2.9.1. Pigment Stability Evaluation

To evaluate the stability of the pigment, the pigment solutions were maintained at pH 6.0 and incubated at different temperatures (30–80 °C) for 0–150 min. The response of pH on pigment stability was studied at a room temperature and different pH values (4.0–8.0) for 0 to 150 min. Sodium citrate–phosphate and sodium phosphate buffers (0.2 M) were used to maintain the pH. The percentage of residual pigment intensity over time was estimated.

#### 2.9.2. Antioxidant Activity of Fungal Pigment

The antioxidant capacity of various concentrations of ethyl acetate extracted fungal pigments (10, 20, 40, 60, 80, and 100 µg/mL) was determined via the phosphomolybdenum assay [41], providing an estimate of the total antioxidant capacity (TAC) by quantifying the reduction in Mo(VI) to Mo(V) and the subsequent formation of a green phosphate Mo(V) complex in an acidic medium. Briefly, the assay mixture was prepared by mixing 0.6 M sulfuric acid, 28 mM sodium phosphate, and 4 mM ammonium molybdate. A 0.1 mL aliquot of each sample, as well as blanks and standards, was mixed with 1 mL of this reagent in test tubes. The samples were heated at 95 °C for 90 min; then, the tubes were taken out and allowed to cool at room temperature before measuring the absorbance at 695 nm, using the blank as a reference. The higher the absorbance value, the stronger the antioxidant activity. All assays were carried out in triplicate, and ascorbic acid was used as the standard for comparison. The scavenging efficiency was computed following the formula given.

$$Antioxidant\;activity\;\left(\%\right)=\left(\frac{Asample-Ablank}{Acontrol-Ablank}\right)\times 100$$

### 2.10. Chitosan Production from Remaining Fungal Biomass Wastes

Chitosan was obtained from the de-oiled fungal biomass left over after biodiesel extraction. The fungal culture was grown on rotted date fruits, the medium after the culture was first used for pigment production, and the fungal biomass collected from the culture was lipid, extracted to get the biodiesel. The leftover biomass after the oil removal was utilized as the raw material for the chitosan extraction, which is considered as the most efficient, sustainable fungal component utilization system. For the beginning of the extraction process, the fungal biomass was dried and then mixed with 0.5 N sodium hydroxide (1:30, *w*/*v*) in a homogenizer and heated at 121 °C for 20 min to remove proteins. The alkali-insoluble material (AIM) obtained was separated by centrifugation at 10,000 rpm for 15 min, extensively washed with distilled water until a neutral pH was reached, dried, and weighed. Dried AIM (1 g) was then processed with 40 mL of 2% acetic acid at 95 °C for 6 h according to the method of Synowiecki and AlKhateeb [42]. After the incubation, the sample was centrifuged at 10,000 rpm for 15 min, and the supernatant containing the solubilized fungal chitosan was taken. Chitosan was recovered as a solid by changing the pH to 10.0 with 2 M NaOH, continuing with a centrifugation at 10,000 rpm for 15 min. The sediment was resuspended in distilled water until pH 7 and then in 95% ethanol (1:20, *w*/*v*), and finally, it was dried at 60 C for 24 h. The yield of purified fungal chitosan was estimated by a gravimetric method, as the alkali, insoluble fraction obtained after sequential alkali and acid extraction generally consists of almost pure mycelial chitosan. The crude yield of chitosan from the remaining fungal biomass was determined by the following formula:

$$Chitosan\;yield\;(\%)=\left(\frac{Dry\;weight\;of\;obtained\;chitosan}{Dry\;weight\;of\;sample}\right)\times 100$$

The total D glucosamine thus obtained was next quantified by dinitrosalicylic acid (DNS) method according to Huet et al. [43]. Infrared Spectroscopy (FTIR) analysis was employed as one of the measures to establish the structural properties of the fungal chitosan that was isolated using a Thermo Scientific Nicolet 6700 (Thermo Fisher Scientific, Inc., Waltham, MA, USA) FTIR spectrometer. The measurements were made in the range of mid infrared from 4000 to 500 cm^−1^. The degree of deacetylation (DD) was estimated according to the baseline method of Domszy and Roberts [44].

### 2.11. Antioxidant Properties of Fungal Chitosan/Pigment Composite

#### 2.11.1. Preparation of Chitosan/Pigment Composite

To prepare the chitosan–fungal pigment composite, 1 mL of fungal pigment (8 mg/mL) was added slowly, drop by drop, to the chitosan solution while stirring continuously, and this step was performed before introducing the sodium tripolyphosphate (TPP) crosslinking agent. Afterward, the resulting gel-like material was allowed to cool, centrifuged to collect the composite, dried overnight at 45 °C, and finally stored at 4 °C.

#### 2.11.2. Characterization of the Chitosan/Fungal Pigment Composite

The encapsulation efficiency (EE%) and loading capacity (LC%) were quantified using UV–visible spectrophotometry at 520 nm, applying the corresponding calculation formulas.

$$EE\;(\%)=\left(\frac{Total\;amount\;of\;Pigment-Free\;Pigment}{Total\;amount\;of\;Pigment}\right)\times 100$$

$$LC\;(\%)=\left(\frac{Total\;amount\;of\;Pigment-Free\;Pigment}{weight\;of\;chitosan}\right)\times 100$$

FTIR spectra were obtained as described previously. The antioxidant capacity of fungal pigments or chitosan or fungal chitosan/pigment composite was determined via the phosphomolybdenum assay as described previously.

#### 2.11.3. Antioxidant Activity of Fungal Chitosan/Pigment Composite

The total antioxidant capacity of fungal pigment, fungal chitosan and fungal chitosan/pigment composite was estimated using the phosphomolybdenum assay [41] as described previously.

## 3. Results

### 3.1. Characterization of Spoilage Date Palm Fruits

Data presented in Table 1 stated that spoilage date palm fruits exhibit a nutrient-rich composition that makes them highly suitable as a low-cost substrate for biomass and lipid production by *Talaromyces atroroseus* QA2602 (PZ091940). Their exceptionally high carbohydrate content, 58% total sugar and 24% reducing sugars, was recorded; additionally, the moderate levels of total soluble proteins (3.1%), free amino acids (1.01%) and 0.21% lipids were estimated. The obtained results revealed the presence of essential minerals—including potassium, magnesium, calcium, and phosphorus recording 0.042, 0.08, 0.035 and 0.079%, respectively. Also, trace elements such as zinc, copper, and cobalt were estimated in spoilage date palm fruits in addition to the relatively low fiber content (2.9%). So, the balanced nutritional profile of spoilage date fruits creates a favorable environment for both fungal growth and lipid accumulation, making them an excellent alternative substrate for microbial lipid biotechnology.

### 3.2. Identification of the Fungal Strain

**Figure 1:**
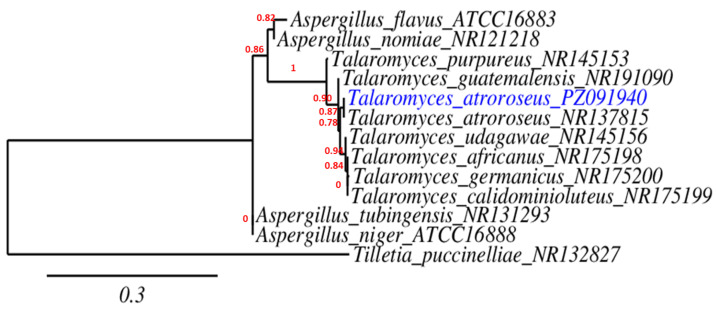
Phylogenetic tree of *Talaromyces atroroseus* PZ091940 with some related fungal species in GenBank database.

The partial 18S rRNA gene sequence (563 bp) of the representative strain *Talaromyces atroroseus* QA2602 (PZ091940) showed 99.12% sequence similarity to *Talaromyces atroroseus* CBS 133442 (NR_137815), indicating a very close phylogenetic relationship between the two taxa (Figure 1).

### 3.3. Lipid Production by Talaromyces atroroseus

Data in Table 2 revealed that increasing spoilage date fruit concentration strengthens lipid production up to an optimum at 100 g/L SDF (58.1 ± 1.8 g/L total sugars), exhibiting the highest lipid titer (3.9 ± 0.3 g/L), lipid content (27.59%), and biomass (14.1 ± 0.4 g/L), whereas lipid yield per spoilage date fruit enhanced by increasing SDF concentration and ranged from 29.6 ± 1.4 to 38.9 ± 3.2 mg/g, indicating robust conversion under moderate carbon levels. Above 100 g/L, performance deteriorates as follows: sugar utilization declines sharply, residual sugars accumulate, and both biomass and lipid titers decrease. This pattern is consistent with substrate/osmotic inhibition, possible inhibitory constituents in SDF at high loadings, oxygen transfer limitations from increased viscosity, and/or nutrient imbalance under extreme carbon excess. Practically, maintaining sugars in the 40–60 g/L range (75–100 g/L SDF in this system) is recommended for maximizing lipid productivity while minimizing residuals. A fed batch strategy that avoids high instantaneous sugar concentrations, with appropriate C/N tuning and oxygen transfer management, should preserve the favorable yields seen at ≤100 g/L SDF. In conclusion, given a discrepancy between measured lipid % and mass-based estimates at some points, confirming the basis/timing of lipid % determinations will improve comparability and scale up confidence.

### 3.4. Effect of Pre-Treatment Method on Lipid Accumulation

The results in Table 3 showed that both temperature and physio-chemical pretreatment method strongly influence sugar release, biomass formation, and lipid accumulation, withphysio-chemicall treatments especially diluting acid, producing the highest values across all recorded parameters. Thermal processing alone increases total sugars and lipids when temperature rises from 50 to 121 °C, but the improvements are modest compared to acid or alkaline treatments. Among all conditions, H_2_SO_4_ at 0.2 M and 121 °C produces the best overall performance, yielding the highest total sugar content (82.4 ± 1.3 g/L), maximum dry weight (17.0 ± 0.2 g/L), and peak lipid concentration (6.5 ± 0.3 g/L) with a lipid content of 38.37%. Acid treatments consistently outperform NaOH in terms of lipid titer and biomass, while NaOH treatment with different concentrations at 121 °C tends to produce slightly higher lipid percentages. Increasing the pretreatment temperature from 50 to 121 °C universally enhances sugar release and lipid formation across all chemical concentrations, confirming that high temperature acid pretreatment most effectively disrupts the substrate and promotes lipid accumulation. Consequently, the obtained data indicated that dilute acid at higher temperature provides the optimal balance of sugar liberation, microbial growth, and lipid production, making it the most suitable pretreatment strategy for maximizing lipid yield.

### 3.5. Biodiesel Production

The fatty acid methyl ester (FAME) profile produced by *Talaromyces atroroseus* QA2602 (PZ091940) is dominated by long-chain C16–C18 fatty acids, with methyl octadecenoate (C18:1; 21.76%), methyl palmitate (C16:0; 20.16%), and methyl linoleate (C18:2; 18.11%) forming the core of the lipid fraction (Table 4), reflecting a typical oleaginous fungal pattern characterized by strong fatty acid synthase activity and active Δ9 and Δ12 desaturation pathways. Saturated fatty acids including C12:0 (8.50%), C14:0 (3.83%), C15:0 (1.84%), C16:0 (20.16%), and C18:0 (8.29%) contribute more than 40% of the total FAMEs, indicating robust de novo synthesis and chain elongation, which are well-documented features of lipid-accumulating fungi. Monounsaturated fatty acids such as C16:1 (1.74%), C18:1 (21.76%), C9:1 (6.34%), and short-chain MUFAs (C10:1 and C7:1) reveal significant desaturase activity, while the substantial proportion of C18:2 confirms strong secondary desaturation capacity typical of *Talaromyces atroroseus*. Minor branched and hydroxy fatty acids—iso-C17:0 (0.94%), OH-C16:0 (4.65%), and branched C20 (0.88%)—highlight the strain’s metabolic versatility. Overall, the FAME composition shows that *Talaromyces atroroseus* produces C16–C18-rich oil with a balanced SFA–MUFA–PUFA distribution, resembling microbial single-cell oils known for applications in biodiesel, oleochemicals, and nutritional lipids, confirming the organism’s strong biotechnological potential.

### 3.6. Characterization of the Produced Biodiesel

The obtained data in Table 5 of the produced biodiesel exhibited a density of 873.8 kg m^−3^, a kinematic viscosity of 4.11 mm^2^ s^−1^, a saponification number of 203.65 mg KOH g^−1^, and an iodine value of 61.18 g I_2_ / 100 g. Additionally, the biodiesel showed a higher heating value of 40.16 MJ kg^−1^ and a cetane number of 60.3, indicating good fuel quality. Against standards, EN 14214 requires ρ = 860–900 kg m^−3^, ν = 3.5–5.0 mm^2^ s^−1^, CN ≥ 51, and IV ≤ 120, while ASTM D6751 requires ν = 1.9–6.0 mm^2^ s^−1^ and CN ≥ 45–47 (no density limit); thus, the *Talaromyces*-derived biodiesel in the current study meets EN 14214 for density, viscosity, CN, and IV and meets ASTM D6751 for viscosity and CN, with HHV typical of B100 (37.5–41 MJ kg^−1^) indicating good energy content per mass.

### 3.7. Pigment Production

**Figure 2:**
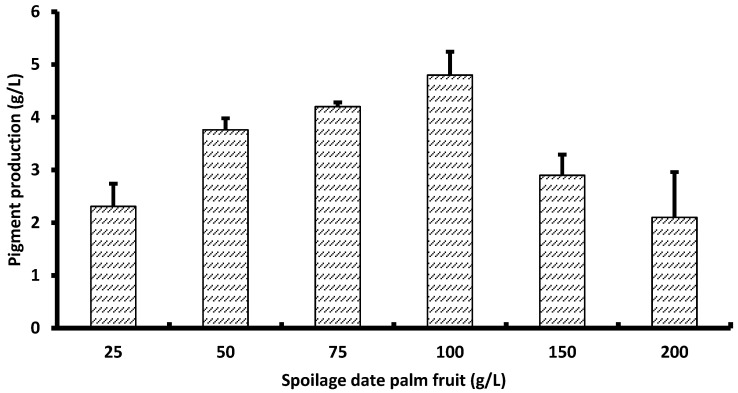
Fungal pigment production from different concentrations of spoilage date fruits.

The obtained data in Figure 2 stated that pigment production rises steadily as spoilage date palm fruit concentration increases from 25 to 100 g/L from 2.31 ± 0.43 to a peak of 4.80 ± 0.44 (more than a twofold gain), indicating that additional carbon in this range stimulates pigment biosynthesis (Figure 2); however, beyond 100 g/L the output drops sharply to 2.90 ± 0.39 at 150 g/L and 2.10 ± 0.86 at 200 g/L, despite the higher substrate supply. This bell-shaped response suggests an optimum near 100 g/L, with the decline in higher loadings likely driven by substrate/osmotic stress, viscosity-related oxygen transfer limitations, and/or inhibitory compounds enriched in the spoilage feedstock at elevated concentrations. Practically, maintaining the effective concentration around 75–100 g/L, for example, via fed-batch feeding should sustain high pigment titers while avoiding the inhibitory effects observed at ≥150 g/L.

Furthermore, the obtained results showed that fungal pigment production increases noticeably with temperature and is further enhanced by thermo-chemical treatment, particularly with dilute sulfuric acid. Thermal treatment alone yielded moderate pigment levels, rising from 4.62 ± 0.42 g/L at 50 °C to 5.72 ± 0.21 g/L at 121 °C, indicating that heat facilitates pigment release or metabolic activation. When combined with H_2_SO_4_, pigment production improved across all concentrations, with the highest yield observed at 0.2 M H_2_SO_4_ at 121 °C (8.35 ± 0.08 g/L), demonstrating a strong synergistic effect of acid hydrolysis and high temperature in breaking down fungal cell walls and enhancing pigment extraction. At 50 °C, acid also improved production, especially at 0.05 M, though the effect was less pronounced than at 121 °C. In contrast, NaOH pretreatment produced moderate enhancement at 50 °C (6.71–6.82) but was less effective or even inhibitory at 121 °C, as seen by the drop to 5.17 ± 0.37 g/L at 0.5 M, suggesting that high-temperature alkali may degrade pigment molecules or negatively alter fungal cell components.

#### 3.7.1. Pigment Characterization

**Figure 3:**
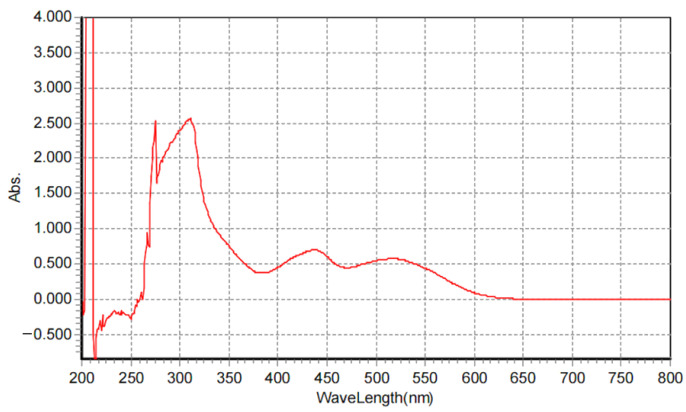
The UV–Vis scan of the obtained fungal pigment.

The UV–Vis scan of the fungal pigment revealed a distinct absorption profile characteristic of conjugated, chromophoric fungal metabolites as shown in Figure 3. The spectrum showed a sharp, intense peak at 204–210 nm, where absorbance reaches the instrument’s upper limit, indicating strong π→π transitions typically associated with highly conjugated molecular structures. Following this region, the absorbance drops and then gradually rises again into a broad, well-defined absorption band spanning 260–320 nm, with a clear maximum around 274–300 nm—a key feature of polyketide-type fungal pigments. The absorbance between 300 and 350 nm remains moderately high, reflecting extended conjugation and aromaticity often reported in *Talaromyces atroroseus* QA2602 (PZ091940) pigments. Beyond 350 nm, the absorption shows a measurable absorbance into the visible range (400–500 nm) at 420 and 520 nm, due to the presence of chromophoric components. Overall, the UV–Vis profile indicates a stable, strongly UV-absorbing pigment with a polyketide-like electronic structure as well the chromophoric components, consistent with reported spectral features of *Talaromyces* secondary metabolites. This pattern supports the pigment’s suitability for applications requiring UV absorption, antioxidant functionality, or chromophoric stability.

#### 3.7.2. GC/Ms Analysis of Fungal Pigments

The GC–MS analysis of the fungal pigment extract (Table 6) showed a chemically diverse mixture dominated by long-chain fatty acid esters and complex nitrogen-containing bicyclic compounds, indicating the presence of both lipophilic carriers and aromatic bioactive metabolites involved in pigment formation. Medium- and long-chain esters such as propionic acid 4-hydroxy-3-hexyl ester (6.90%), 3-methyl-2-butenoic acid 4-hexadecyl ester (12.81%), and butyric acid 4-pentadecyl ester (4.16%) reflect active lipid metabolism and suggest hydrophobic matrices that stabilize or transport pigments. Volatile acid derivatives like 2-methylbutanoic anhydride (10.49%) and oxygenated esters such as 1-propoxypropan-2-yl 3-methylbutanoate (9.07%) point to branched-chain amino acid catabolism and secondary metabolic modifications commonly associated with fungal pigment biosynthesis. The presence of structurally complex heterocyclic molecules—particularly 2,5-dimethyl-7,7-diphenyl-3-aza-4,6-dioxabicyclo [3.2.0]hept-2-ene (1.34%) and the dominant compound, 8-azabicyclo[3.2.1]octan-3-ol with a diphenylethyl substituent (42.33%)—indicates formation of aromatic and alkaloid-like chromophoric structures likely responsible for the pigment’s color intensity and bioactivity. Thus, the chemical pattern suggests a pigment system composed of both lipid-derived esters and nitrogenous aromatic compounds, reflecting a complex fungal secondary metabolism with potential antimicrobial, antioxidant, or industrially relevant properties.

#### 3.7.3. Pigment Stability

**a.**  **pH stability**

**Figure 4:**
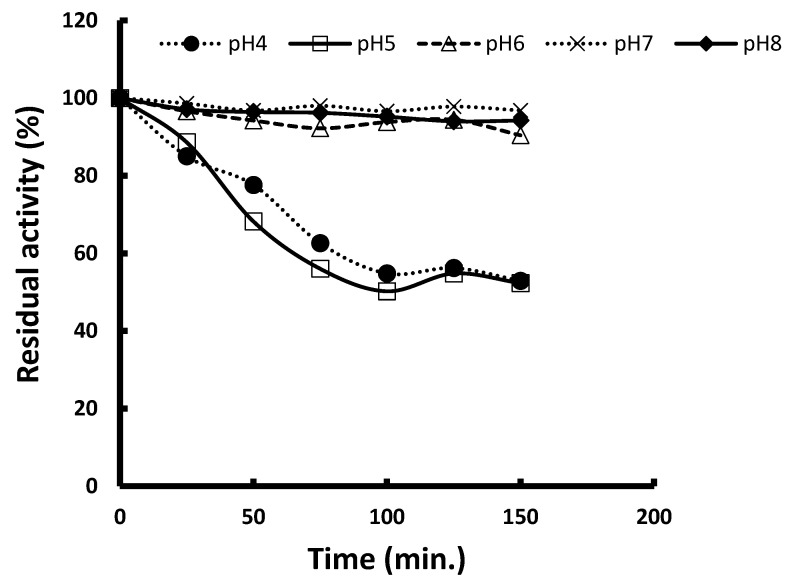
pH stability of fungal pigment produced by *Talaromyces atroroseus* PZ091940.

The pH stability profile of the fungal pigment in Figure 4 showed that fungal pigment is highly sensitive to acidic conditions but remains remarkably stable from neutral to mildly alkaline pH levels. At pH 4 and 5, the pigment undergoes rapid degradation, with retention dropping to nearly half of the original intensity by 150 min, indicating that the pigment is acid-labile and structurally unstable in low pH environments. In contrast, at pH 6, 7, and 8, the pigment retains more than 90% of its initial intensity throughout the entire 150 min period, demonstrating excellent stability and minimal degradation under neutral and alkaline conditions. Overall, the pigment exhibits its lowest stability in acidic media and its highest stability between pH 6 and 8, making neutral to slightly alkaline formulations the most suitable for maintaining pigment integrity in applied uses such as food, cosmetic, or pharmaceutical systems.

**b.**  **Thermal stability**

**Figure 5:**
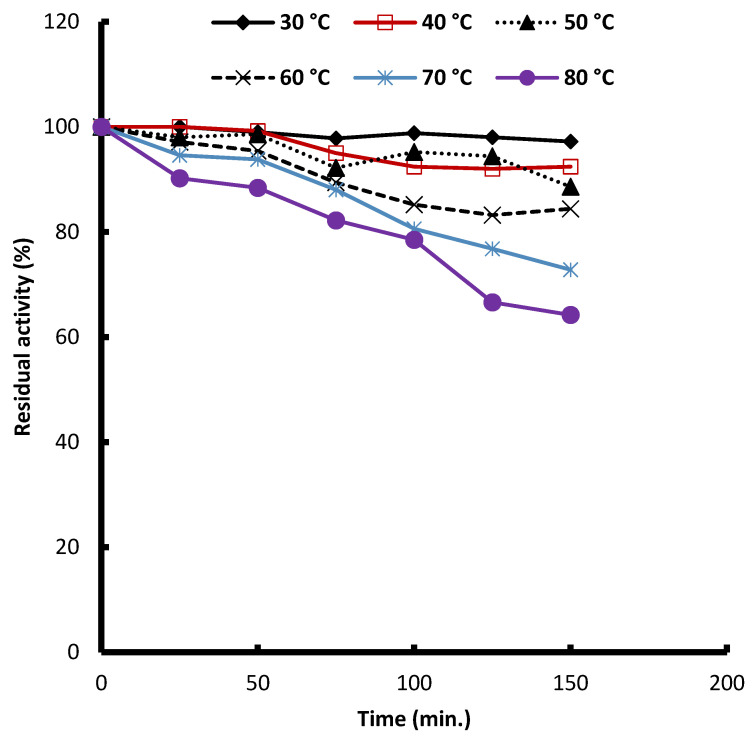
Thermal stability of *Talaromyces atroroseus* pigments.

The fungal pigment demonstrates excellent thermal stability at low to moderate temperatures but shows progressive degradation at higher temperatures, particularly above 60 °C (Figure 5). At 30 °C and 40 °C, pigment intensity remains essentially unchanged over the full 150 min period, indicating outstanding stability under typical storage or processing temperatures. At 50 °C and 60 °C, only slight reductions are observed, with pigment retention remaining above 85–98%, reflecting good tolerance to moderate heat exposure. However, at 70 °C and especially at 80 °C, thermal degradation becomes more pronounced: pigment retention declines steadily from 94 to 100% at time zero to about 73% at 70 °C and only 64% at 80 °C after 150 min. These results show that while the pigment is highly stable up to 60 °C, stability significantly decreases at ≥70 °C, suggesting that prolonged exposure to high temperatures accelerates pigment breakdown. Accordingly, the pigment is suitable for applications that involve mild to moderate heat but may require protective strategies or lower temperature processing for high temperature industrial uses.

#### 3.7.4. Antioxidant Activity of Fungal Pigments

**Figure 6:**
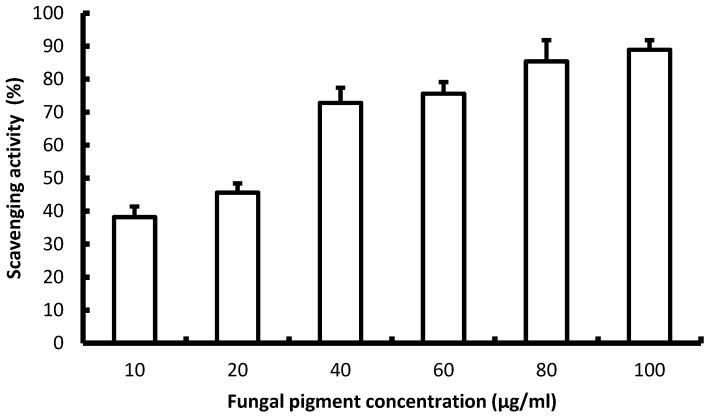
Total antioxidant capacity of *Talaromyces atroroseus* PZ091940 fungal pigments measured using a phosphomolybdenum technique.

The pigment showed a clear dose-dependent increase in scavenging activity from 38.2 ± 3.2% (10 µg/mL) to 88.9 ± 2.9% (100 µg/mL), with a steep response between 20 and 40 µg/mL (45.6 to 72.8%), indicating a potent mid-range effect as shown in Figure 6. By linear interpolation, the EC_50_ is 23.6 µg/mL, reflecting high antioxidant potency at relatively low doses. Above 60 µg/mL, the curve approaches a plateau (75.6–88.9% from 60 to 100 µg/mL), suggesting near maximal quenching capacity is reached by 80–100 µg/mL. Thus, the obtained data supported that the pigment is an effective radical scavenger, with strong activity above 40 µg/mL and near saturation by 80–100 µg/mL.

### 3.8. Chitosan Production from De-Oiled Fungal Biomass

**Figure 7:**
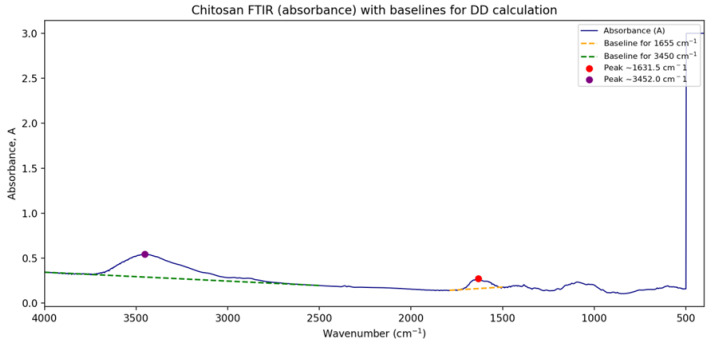
The FTIR graph of fungal chitosan used to determine the degree of deacetylation (DD).

The results obtained showed that the chitosan yield from de-oiled fungal biomass represented 7.82% of de-oiled fungal dry biomass, and glucosamine content of extracted fungal chitosan was estimated by 81.09% of extracted chitosan. The IR spectroscopic method is used to determine the degree of deacetylation (DD) value of mycelial chitosan as shown in Figure 7. The DD value of fungal chitosan obtained from *Talaromyces* de-oiled fungal biomass was 67.6%. The FTIR spectrum of the extracted chitosan showed the characteristic functional groups expected for a well-defined chitosan structure. The broad, intense band at 3451 cm^−1^ corresponds to overlapping O–H and N–H stretching vibrations, indicating the presence of hydrogen bonded hydroxyl and amine groups typical of chitosan. The peaks observed around 1630 cm^−1^ are attributed to amide I (C=O stretching) or N–H bending, reflecting residual acetylated units, while the band near 1383 cm^−1^ corresponds to C–H bending of the polysaccharide backbone. Additionally, the peak around 1323 cm^−1^ represents C–N stretching, confirming the presence of amino groups associated with the deacetylated structure of chitosan. Altogether, these absorption bands align well with standard chitosan profiles, supporting the successful extraction and structural integrity of the polymer.

### 3.9. Antioxidant Properties of Fungal Chitosan/Pigment Composite

#### 3.9.1. Chitosan/Pigment Composite Characterization

The fungal chitosan–pigment composite exhibited an encapsulation efficiency (EE%) of 74% and a loading capacity (LC%) of 23%, indicating that a substantial amount of the fungal pigments was effectively incorporated into the fungal chitosan matrix. These values reflect successful entrapment and suggest good formulation performance.

**Figure 8:**
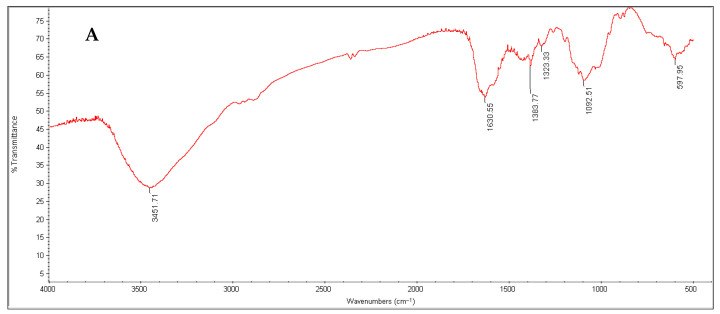

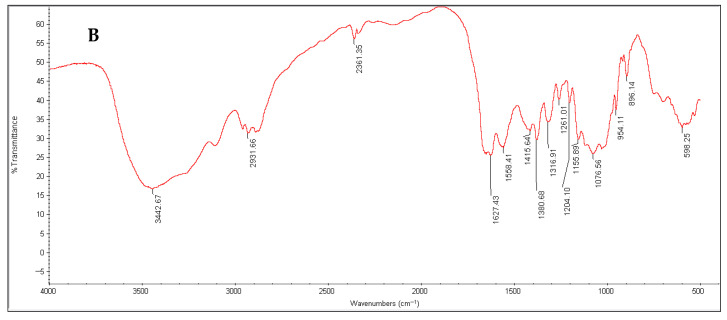
FTIR spectra of fungal chitosan (**A**) and fungal chitosan/pigment composite (**B**).

The FTIR spectra of fungal chitosan and the chitosan/pigment composite revealed several important differences that highlight how pigment incorporation alters the chemical environment of the chitosan matrix (Figure 8 and Table 7). In the fungal chitosan spectrum, the broad band at 3451 cm^−1^ corresponds to O–H and N–H stretching vibrations typical of hydrogen-bonded polysaccharides, whereas in the composite this band shifts to 3413 cm^−1^, indicating strengthened hydrogen bonding due to interactions between chitosan chains and the incorporated pigment. The amide I region also shows a slight shift from 1631 cm^−1^ in fungal chitosan to 1627 cm^−1^ in the composite, suggesting changes in the local environment of residual acetyl groups, possibly through π–π or hydrogen bond interactions with pigment molecules. Additional bands that appear or become more pronounced in the composite including those at 1415, 1281, and 1261 cm^−1^ that represent CH_2_ bending and C–N or C–O vibrations, indicating structural changes within the chitosan backbone influenced by pigment binding.

Furthermore, while fungal chitosan shows a strong polysaccharide C–O–C vibration at 1029 cm^−1^, this band shifts to around 986 cm^−1^ in the composite, a notable movement that implies alterations in the pyranose ring environment or modifications in hydrogen bonding networks. The composite also exhibits additional low-frequency skeletal vibrations at 668 cm^−1^ and 589 cm^−1^ not listed for the fungal chitosan. These lower wavenumber bands may reflect pigment-associated ring deformation or aromatic C–H out-of-plane contributions, further supporting the conclusion that the pigment interacts intimately with the chitosan structure. So, the comparison shows that pigment incorporation leads to measurable shifts in chitosan’s characteristic absorption bands, introduces new vibrational features, and strengthens hydrogen bond interactions—confirming successful formation of a chitosan/pigment composite with modified structural and chemical properties.

#### 3.9.2. Antioxidant Properties of Fungal Pigments, Chitosan and Fungal Chitosan/Pigment Composite

**Figure 9:**
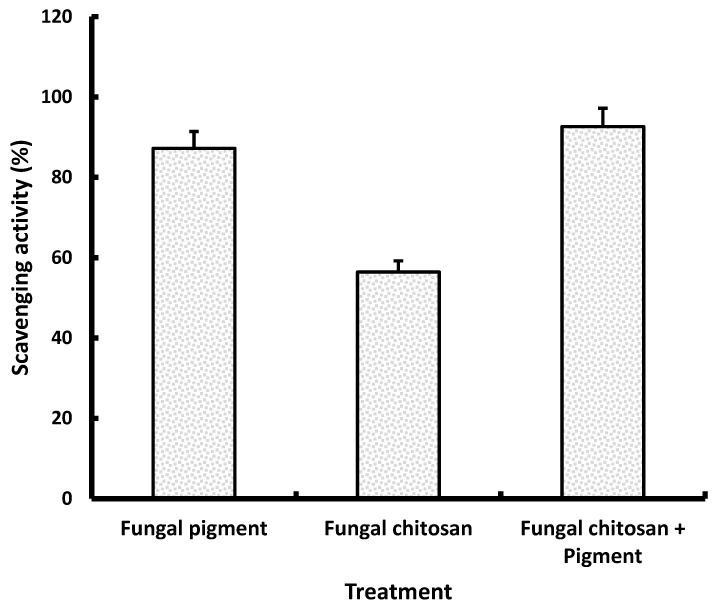
Antioxidant properties of fungal pigments, chitosan and fungal chitosan/pigment composite.

The antioxidant assay demonstrates that the fungal pigment alone exhibits strong radical scavenging activity (87.2 ± 4.2%), confirming its high intrinsic antioxidant potential (Figure 9). In comparison, fungal chitosan shows a much lower activity (56.4 ± 2.8%), indicating that although chitosan contributes to antioxidant defense, it is considerably less effective than the pigment. Notably, the combination of fungal chitosan and pigment results in the highest scavenging activity (92.6 ± 4.6%), exceeding the effects of either component alone. This suggests a synergistic or additive interaction, where chitosan may enhance pigment stability, solubility, or accessibility to free radicals. So, these findings indicated that while the pigment is the primary antioxidant agent, its combination with chitosan offers superior scavenging capacity and may be advantageous for developing antioxidant rich bioproducts.

## 4. Discussion

The utilization of rotten date fruits as multifunctional feedstock is an environmentally friendly way to produce biodiesel, natural pigments, and fungal chitosan in a biorefinery system. Having highly fermentable sugar content, spoiled (or rotting) date fruits are recognized very well as efficient and cheap substrates for microbial lipid production; thus, they can be easily converted to single cell oils for biodiesel, as has been demonstrated in waste valorization research [24]. Oleaginous fungi like *Talaromyces* can metabolize these sugars into C16–C18 fatty acids, which are very suitable for the synthesis of biodiesel; therefore, microbial lipids are compositionally quite similar to vegetable oils, as confirmed by several studies [45]. Simultaneously, *Talaromyces* species produce natural pigments abundantly, with the metabolite and pigment production being highly influenced by the choice of substrate, namely agricultural waste [46,47]. After the lipids have been extracted, the fungal biomass from which the oil has been obtained can be used as a starting material for the production of chitosan, a method that is consistent with the results of fungal, chitosan characterization studies which not only highlight the high purity but also the favorable functional properties from fungal sources [48]. These technologies combined highlight the potential of spoiled date fruits serving as a multipurpose feedstock that supports fungal production of biodiesel precursors, pigments, and chitosan in one efficient and environmentally friendly production system.

The compositional analysis of spoilage date palm fruits demonstrates that they are highly suitable as a low-cost substrate for biomass and lipid production by *Talaromyces atroroseus*. Their high carbohydrate content, reflected in substantial levels of fermentable sugars, aligns with previous findings showing that sugar-rich agro-industrial residues provide the carbon flux necessary to support rapid fungal growth and lipid biosynthesis [49]. Date waste valorization emphasizes that date fruits contain abundant simple sugars that can be efficiently converted into microbial lipids, consistent with observations in oleaginous fungi grown on similar waste streams. Likewise, broader analyses of microbial lipid production confirm that carbon rich substrates favor high lipid accumulation by driving acetyl CoA supply and fatty acid synthesis pathways [50,51]. The moderate nitrogen content derived from soluble proteins and free amino acids supports early biomass formation while permitting nitrogen limitation during later culture stages, a key trigger for lipid accumulation in oleaginous fungi, as widely reported in microbial lipid physiology [52].

The lipid production profile obtained from *Talaromyces atroroseus* cultivated on spoilage date fruit (SDF) shows lipid synthesis increased steadily with SDF concentration and reached its optimum at 100 g/L SDF, corresponding to 58.10 ± 1.83 g/L total sugars, where the fungus achieved its highest lipid titer (3.89 ± 0.32 g/L), lipid content (27.59%), and biomass production (14.1 ± 0.44 g/L). This pattern aligns with broader observations in microbial lipid systems, where moderate carbon excess stimulates both cell proliferation and lipid accumulation, as documented in large-scale reviews of waste-based lipid bioprocessing [50]. The increase in lipid yield per gram of spoilage date fruit (29.6 ± 1.42–38.9 ± 3.24 mg/g) further confirms efficient substrate conversion under these carbon levels, consistent with findings that sugar-rich agricultural residues serve as highly effective feedstocks for single cell oil production [24]. However, beyond the 100 g/L SDF threshold, system performance began to decline. This decline manifested as reduced sugar utilization, accumulation of residual sugars, and decreased biomass and lipid titers, indicators of substrate inhibition and metabolic stress. Similar inhibition at high substrate concentrations is widely reported in lipid-producing fungi and is attributed to factors such as osmotic stress, buildup of inhibitory compounds present in agrowaste hydrolysates, and oxygen transfer limitations caused by increased medium viscosity [50]. Excessive carbon can also disrupt the delicate C/N balance necessary for lipid biosynthesis, as nitrogen depletion must coincide with a metabolically manageable carbon load to direct flux toward storage lipids rather than stress responses [24]. To sustain high lipid yields during scale up, fed batch strategy is particularly advantageous. Fed batch feeding prevents spikes in sugar concentration, thus reducing osmotic stress and limiting the formation of inhibitor compounds during hydrolysis. The obtained data confirm spoilage date fruit as a highly effective, low-cost substrate for *Talaromyces atroroseus* lipid production and reinforce the importance of carbon moderation and controlled feeding strategies in achieving maximum lipid productivity.

The effect of thermal and thermo-chemical pretreatments demonstrates that substrate accessibility is the primary driver of reducing sugar release and subsequent microbial productivity. The marked superiority of thermo-chemical pretreatment particularly dilutes sulfuric acid hydrolysis, aligns with the extensive literature showing that acid pretreatment is the most effective approach for solubilizing complex fruit-based substrates and maximizing fermentable sugar yield. In the present study, dilute H_2_SO_4_ at 0.2 M and 121 °C yielded the highest reducing sugar concentration (82.41 ± 1.3 g/L), along with the maximum biomass (17.02 ± 0.16 g/L) and lipid accumulation (6.53 ± 0.31 g/L, 38.37%). Dilute acid treatment substantially enhances the release of simple sugars from date-derived materials by breaking down hemicellulosic components and weakening cell wall integrity, thereby improving downstream microbial conversion efficiency. Similarly, Khorshidian et al. [24] highlight that high temperature acid pretreatment is one of the most reliable strategies for achieving both rapid saccharification and improved bioconversion of date waste into value-added products such as organic acids, lipids, and biofuels [26]. The universal improvement in performance with rising temperature (50 to 121 °C) across all chemical concentrations reinforces the mechanistic role of heat in disrupting substrate structure and enhancing hydrolysis kinetics. High temperature processing increases diffusion rates, weakens biomass rigidity, and improves pore accessibility conditions that strongly promote both carbohydrate solubilization and microbial conversion efficiency [24].

The fatty acid methyl ester (FAME) composition obtained from *Talaromyces atroroseus* reflects a lipid profile highly suitable for biodiesel applications. The predominance of C16–C18 fatty acids, particularly methyl oleate (C18:1; 21.76%), methyl palmitate (C16:0; 20.16%), and methyl linoleate (C18:2; 18.11%), is consistent with the characteristic signatures of oleaginous fungi described in broader microbial lipid producers. Such organisms typically accumulate long-chain fatty acids similar to those found in vegetable oils, making their single cell oils attractive substitutes for biodiesel feedstocks [12]. The strong representation of both saturated and unsaturated C16–C18 FAMEs aligns with the metabolic behavior of lipid-accumulating fungi, which often rely on active fatty acid synthase pathways for de novo synthesis and utilize Δ9 and Δ12 desaturases to generate monounsaturated and polyunsaturated fatty acids, respectively [52]. The substantial proportion of saturated fatty acids, namely, C12:0 (8.50%), C14:0 (3.83%), C15:0 (1.84%), C16:0 (20.16%), and C18:0 (8.29%), suggests robust fatty acid chain elongation and efficient conversion of acetyl CoA into higher chain length lipids. These traits are typical of oleaginous fungi and have been documented across various genera, including *Mortierella*, *Umbelopsis*, and *Aspergillus* [45,53]. So, the *Talaromyces*-derived FAME profile with its balanced mix of saturated, monounsaturated, and polyunsaturated fatty acids strongly resembles the composition of established microbial single cell oils used for biodiesel. Long-chain fatty acids, particularly C16–C18, are essential for producing biodiesel with acceptable cetane number, viscosity, and cold flow properties. Studies on fungal oils consistently emphasize that oleaginous fungi represent a renewable and scalable alternative to plant-based oils for biodiesel production due to their comparable FAME distributions and the ability to utilize low-cost substrates [45]. Taken together, the obtained data confirm *Talaromyces atroroseus* as a promising microbial platform for biodiesel, oleochemical, and nutraceutical lipid production, offering both compositional suitability and biotechnological potential.

The obtained physicochemical properties of the *Talaromyces*-derived biodiesel recorded that the density (ρ = 873.8 kg m^−3^), kinematic viscosity (ν = 4.11 mm^2^ s^−1^), saponification number (SN = 203.65 mg KOH g^−1^), iodine value (IV = 61.18 g I_2_/100 g), higher heating value (HHV = 40.16 MJ kg^−1^), and cetane number (CN = 60.3) indicated a high-quality biodiesel consistent with international fuel specifications. When benchmarked against biodiesel specifications, the calculated values fall comfortably within the EN 14214 requirements, which specify density = 860–900 kg m^−3^, ν = 3.5–5.0 mm^2^ s^−1^, CN ≥ 51, and IV ≤ 120. These values also satisfy ASTM D6751, which requires ν = 1.9–6.0 mm^2^ s^−1^ and CN ≥ 45–47. The predicted HHV of 40.16 MJ kg^−1^ aligns with typical heating values for neat biodiesel (B100), which commonly fall between 37.5 and 41 MJ kg^−1^, making this Fungal-derived fuel energetically competitive with conventional biodiesel based on vegetable oils. Moreover, the moderate iodine value (IV = 61) indicates a balanced level of unsaturation—low enough to support oxidative stability yet high enough to maintain favorable cold flow and combustion properties [54]. Such a balanced FAME profile is desirable in biodiesel formulation, as documented in biodiesel property specifications. Viscosity (ν = 4.11 mm^2^ s^−1^) falls neatly within both standards, indicating fuel with good atomization and lubricity, properties essential for injection-based combustion systems. Likewise, the density (ρ = 873.8 kg m^−3^) is well within EN 14214 limits, suggesting compatibility with diesel injection calibration and volumetric energy expectations. Thus, the physicochemical profile of *Talaromyces*-derived biodiesel supports its suitability for bending with petroleum diesel, provided that remaining routine parameters, for example, oxidation stability, metal content, glycerides, and cold flow performance are confirmed in accordance with EN 14214 and ASTM D6751. These additional properties are standard QA/QC requirements for any biodiesel intended for commercial deployment.

Pigment production by *Talaromyces atroroseus* increased steadily as spoilage date palm fruit (SDF) concentration rose from 25 to 100 g/L, where pigment yield more than doubled (from 2.31 to 4.80), indicating that moderate increases in available carbon stimulate secondary metabolite biosynthesis. A similar carbon-dependent enhancement of pigment formation has been reported in *Talaromyces* and related filamentous fungi, where increasing substrate availability within an optimal range promotes intensified metabolic flux toward polyketide-derived pigments [47]. However, as SDF concentration exceeded 100 g/L, pigment output dropped sharply to 2.90 ± 0.39 at 150 g/L and 2.10 ± 0.86 at 200 g/L despite the higher substrate supply. Excessive carbon loading leads to environmental and physiological stress, reduced metabolic efficiency, and suppression of secondary metabolism [55]. The observed decline at ≥150 g/L is likely driven by multiple inhibitory factors. First, osmotic stress at high sugar concentrations is well recognized to hinder fungal metabolic activity and pigment biosynthesis [47]. Second, increased medium viscosity reduces oxygen transfer, an essential component for polyketide pigment biosynthesis mechanics in *Talaromyces*. Studies of pigment-producing *Talaromyces* strains showed that reduced aeration significantly diminishes pigment formation due to impaired oxidative steps in the biosynthetic pathway [55]. Third, spoilage date fruit at high loadings may introduce elevated levels of inhibitory compounds, such as phenolics or organic acids, which can accumulate during thermal or chemical pretreatment and are known to suppress fungal metabolism [46]. Based on these restrictions, pigment production appears to be optimal at 100 g/L SDF, where nutrient availability and physiological conditions align to support efficient secondary metabolism. For practical bioprocessing, maintaining the substrate concentration between 75 and 100 g/L is advisable to sustain high pigment titers while avoiding inhibitory effects. A fed batch feeding strategy would be particularly effective, allowing carbon to be supplied gradually without creating osmotic or rheological burdens, an approach widely recommended for improving yields of fungal metabolites under high substrate systems [47,55].

The obtained data confirmed that *Talaromyces atroroseus* pigments exhibit minimum stability under acidic conditions but maximum stability between pH 6 and 8, where structural integrity and chromophore functionality remain largely intact. From an application standpoint, this indicates that neutral to slightly alkaline formulations such as those used in many food coatings, cosmetic emulsions, and pharmaceutical suspensions are most suitable for maintaining pigment quality and color longevity. This behavior is consistent with broader assessments of *Talaromyces* pigments, which have been noted for their excellent performance in textile and food-related environments when pH is properly controlled [47]. Acid-induced pigment breakdown has been attributed to proton-mediated structural disruption of chromophore groups, making fungal pigments susceptible to color loss in food or processing environments with low pH [56]. Morales-Oyervides et al. [47] reported that the red pigment secreted by *P. puropurogenum* GH2 is found to be stable at temperatures up to 80 °C in the pH range of 4–8, while the red pigment produced by *M. ruber* is stable at lower temperatures ranging from 30 to 60 °C at pH 6–8 [57]. In conclusion, strong pH-dependent stability emphasizes the importance of avoiding acidic processing conditions, while leveraging neutral to mildly alkaline pH ranges to preserve pigment integrity during industrial utilization.

Taken together, the data indicate that the fungal pigment exhibits excellent thermal stability up to 60 °C, enabling its use in food, cosmetic, and pharmaceutical applications involving mild processing, warm storage, or moderate heating. However, temperatures ≥70 °C pose a significant risk of degradation, especially under extended exposure. This suggests that high temperature industrial uses, for example, pasteurization, baking, or high temperature extrusion, may require either protective formulation strategies (e.g., encapsulation, antioxidants, and polymer matrix stabilization) or process adjustments that limit thermal stress. The thermal stability profile of the fungal pigment indicates strong resistance to degradation at low and moderate temperatures but increases susceptibility to breakdown at elevated temperatures. At 30 °C and 40 °C, the pigment remains essentially unchanged over the entire 150 min period, demonstrating excellent retention and stability under typical handling, storage, and mild processing conditions. This agrees with earlier findings on *Talaromyces purpurogenus* pigments, which show high thermal tolerance under moderate heat exposures [58,59]. Similar observations were reported by Ugwu et al. [60], who found that *T. purpurogenus* pigments retained over 80% of their color intensity after exposure to elevated temperatures during submerged and alternating air–liquid phase cultivation. This reinforces the notion that *Talaromyces* pigments are generally stable up to intermediate temperature thresholds. However, thermal degradation becomes increasingly pronounced at ≥70 °C. At 70 °C, pigment retention progressively declines to 73% by 150 min, and at 80 °C, retention drops to approximately 64% over the same period. These results are consistent with prior studies noting that *T. purpurogenus* pigments are heat-stable only up to a certain limit, beyond which structural decomposition accelerates, particularly during prolonged exposure [58]. High temperature degradation is commonly attributed to chromophore destabilization and pigment oxidation, processes known to intensify rapidly above 60–70 °C in natural fungal colorants [60].

The dose-dependent antioxidant response of the fungal pigment shows a clear and progressive increase in radical scavenging activity between 10 and 100 µg/mL, rising from 38.2 ± 3.2% to 88.9 ± 2.9%. This sharp increase, particularly the pronounced jump between 20 and 40 µg/mL (45.6 to 72.8%), indicates a potent mid-range antioxidant effect, consistent with reports that *Talaromyces atroroseus* pigments exhibit strong redox-active properties due to their polyketide-derived chromophores. Studies on *Talaromyces* pigments, such as those by Morales Oyervides et al. [47], similarly document high antioxidant activity across fungal pigment extracts and emphasize their potential for food and cosmetic applications. Additional work on *Talaromyces purpurogenus* also confirms that its pigments possess notable antioxidant capacity, including free radical quenching effects in both aqueous and organic phases [60]. Based on linear interpolation, the estimated EC_50_ of 23.6 µg/mL indicates that the pigment achieves half maximal scavenging activity at relatively low concentrations, an indicator of high antioxidant potency when compared with many natural pigments and phenolic-based systems. The curve begins to level off above 60 µg/mL, reaching a near plateau between 75.6% and 88.9% from 60 to 100 µg/mL. This saturation behavior suggests that most reactive sites are occupied by 80–100 µg/mL, beyond which further increases in pigment concentration produce only marginal gains in scavenging activity. Similar saturation kinetics are reported for fungal pigments in the literature, where maximal quenching often occurs within a comparable concentration range [47,60].

The extraction of chitosan from *Talaromyces* de-oiled fungal biomass yielded 7.82% (*w*/*w*) chitosan relative to the dry biomass, a value that falls within the expected range reported for fungal chitosan obtained from filamentous fungi grown on nutrient-rich substrates. Fungal-derived chitosan yields are known to vary widely depending on species, growth conditions, degree of cell wall maturation, and efficiency of deproteinization and deacetylation steps. For example, Sousa et al. [48] reported that fungal chitosan extracted through sequential demineralization, deproteinization, and deacetylation exhibits yield variability reflecting differences in fungal cell wall composition and processing parameters. Thus, the measured yield of 7.82% indicates an efficient recovery process that is comparable to other well-optimized fungal chitosan extraction systems. The degree of deacetylation (DD), a critical determinant of chitosan’s physicochemical and functional properties, was calculated to be 67.6%, based on IR spectroscopic analysis. This value places the material within the medium DD category, which is known to confer good solubility in dilute acids, favorable film forming behavior, and useful antimicrobial activity. FTIR-based DD estimation is widely adopted for fungal chitosan characterization because diagnostic peaks corresponding to N–H bending, amide group vibrations, and C–N stretching reliably track the extent of acetyl removal. As reported by Sousa et al. [48], the presence of characteristic bands around 3400 cm^−1^ (O–H/N–H stretching), 1650 cm^−1^ (amide I), and 1320–1380 cm^−1^ (C–N/C–H bending) is typical of properly deacetylated fungal chitosan and is used to derive DD values comparable to those obtained from the current study.

The antioxidant assay showed that the fungal pigment possesses inherently strong radical scavenging capacity, achieving 87.2 ± 4.2% activity, which aligns with earlier reports describing *Talaromyces* metabolites as rich in bioactive compounds, including pigments known for their antioxidant potential. Adelusi et al. [44] demonstrated that *Talaromyces pinophilus* produces several metabolites, including fatty acids and aromatics, with documented antioxidant properties, supporting the high intrinsic activity observed for the pigment alone. In contrast, fungal chitosan exhibits moderately lower scavenging activity (56.4 ± 2.8%), consistent with the literature noting that chitosan contributes to antioxidant defense primarily through proton donating and metal chelating mechanisms but is typically less potent than pigment-based antioxidants. Furthermore, the obtained data revealed the markedly higher antioxidant activity of the chitosan–pigment composite (92.6 ± 4.6%), which exceeds that of either component alone. This enhancement suggests a synergistic or additive interaction, likely arising from improved pigment stability, enhanced dispersion, or increased accessibility of active chromophores when embedded in a chitosan matrix. Similar synergetic effects have been highlighted in stability studies of *Talaromyces* pigments, where the surrounding matrix significantly influences color retention and functional performance under environmental stressors [58]. Moreover, the composite mechanism reflects patterns observed in microbial pigment systems, where carrier materials enhance the bioactivity and stability of pigment molecules [47].

## 5. Conclusions

The current study demonstrated that spoilage date palm fruits constitute a robust, low-cost feedstock for integrated bioprocessing with *Talaromyces atroroseus*. Their sugar-rich profile (high total and reducing sugars) with supportive levels of proteins, amino acids, minerals, and low fiber provides a balanced medium that promotes fungal growth, lipid accumulation, and pigment biosynthesis. Process optimization revealed that 100 g L^−1^ SDF is the practical optimum for lipid production; higher loadings impair performance, consistent with osmotic/substrate inhibition, increased viscosity, and oxygen transfer limits. Dilute-acid thermo-chemical pretreatment (0.2 M H_2_SO_4_, 121 °C) maximized sugar liberation, biomass, and lipid titer, establishing a clear pretreatment window for scale-up. The resulting oil, enriched in C16–C18 FAMEs, yielded biodiesel whose density, viscosity, iodine value, and cetane number satisfy EN 14214 and ASTM D6751 criteria, confirming strong fuel quality. In parallel, pigments displayed optimal production at ~100 g L^−1^ SDF, enhanced by acid-assisted heat treatment, and exhibited UV–Vis features of polyketide-like chromophores, favorable pH (6–8) and thermal (≤60 °C) stability, and potent antioxidant activity (low EC_50_). Additionally, fungal chitosan recovered from de-oiled biomass (DD 67.6%) and its chitosan–pigment composite showed clear spectroscopic interactions, enabling value-added co-products. Consequently, these findings validate spoilage dates as a circular-bioeconomy substrate for concurrent biodiesel, pigments, and chitosan production using *T. atroroseus*.

## Figures and Tables

**Table 1:** The physicochemical composition of the spoilage date palm fruit.

Constituent	Value (%)
Total sugar	62
Reducing sugars	21
Total soluble proteins	3.1
Total lipids	0.21
Free amino acids	1.01
Sodium	0.005
Potassium	0.042
Calcium	0.035
Magnesium	0.08
Phosphorus	0.079
Zinc	0.003
Cobalt	0.006
Copper	0.004
Fiber	2.9

**Table 2:** Lipid production by *Talaromyces* cultivated on different concentrations of spoilage date palm fruit.

Spoilage Date Palm Fruit Concentration (g/L)	Total Sugar Content (g/L)	Dry Weight (g/L)	Lipid Conc. (g/L)	Lipid Content (%)	Residual Sugar (g/L)	Consumed Sugar (g/L)	Lipid Yield (mg Lipid/g Spoilage Date)
25	13.6 ± 1.0	5.4 ± 0.2	0.7 ± 0.1	13.7	6.3 ± 0.2	7.3 ± 0.3	29.6 ± 1.4
50	27.2 ± 0.8	9.2 ± 0.4	1.7 ± 0.4	18.8	9.5 ± 0.2	17.7 ± 1.4	34.6 ± 3.6
75	39.8 ± 2.1	11.5 ± 0.6	2.5 ± 0.7	21.4	14.9 ± 0.4	24.8 ± 2.3	32.8 ± 2.4
100	58.1 ± 1.8	14.1 ± 0.4	3.9 ± 0.3	27.6	29.0 ± 1.0	29.1 ± 0.7	38.9 ± 3.2
150	93.1 ± 3.2	12.0 ± 0.5	2.9 ± 0.4	24.2	47.1 ± 0.8	46.0 ± 4.2	19.4 ± 0.8
200	116.4 ± 2.0	8.5 ± 0.2	1.4 ± 0.2	15.9	78.3 ± 2.6	38.1 ± 2.3	6.8 ± 0.2

**Table 3:** Effect of chemical, thermal and physio-chemical treatment on reducing sugar concentration, lipid and pigment production.

Treatment Method	Chemical Agent		Temperature (°C)	Total Sugar Content	Dry Weight (g/L)	Lipid Concentration (g/L)	Lipid Content (%)	Pigment Production (g/L)
	Type	Conc (M)						
Thermal			50	43.9 ± 1.2	11.5 ± 2.0	2.7 ± 0.1	23.7	4.6 ± 0.4
			121	56.6 ± 0.8	8 ± 2.3	3.9 ± 0.2	28.3	5.7 ± 0.2
Thermo-chemical	H_2_SO_4_	0.05	50	49.4 ± 1.1	15.5 ± 1.0	5.2 ± 0.3	33.3	7.1 ± 0.1
			121	63.1 ± 0.9	16.5 ± 0.4	5.4 ± 0.1	32.5	7.9 ± 0.1
		0.1	50	55.6 ± 0.3	12.1 ± 0.2	4.0 ± 0.3	32.8	6.4 ± 0.2
			121	71.9 ± 1.4	16.1 ± 0.0	5.4 ± 0.2	33.5	7.3 ± 0.2
		0.2	50	64.5 ± 2.1	16.0 ± 0.2	5.4 ± 0.1	34.0	6.9 ± 0.4
			121	82.4 ± 1.3	17.0 ± 0.2	6.5 ± 0.3	38.4	8.4 ± 0.1
	NaOH	0.5	50	61.1 ± 0.9	14.8 ± 0.4	3.6 ± 0.1	24.7	6.7 ± 0.1
			121	65.0 ± 0.9	13.9 ± 0.5	3.8 ± 0.1	27.5	5.2 ± 0.4
		1.0	50	60.9 ± 1.2	15.1 ± 0.3	4.2 ± 0.4	27.9	6.8 ± 0.1
			121	70.4 ± 0.5	14.6 ± 0.4	4.7 ± 0.1	31.98	6.5 ± 0.2
		1.5	50	63.0 ± 2.2	14.7 ± 0.2	4.5 ± 0.2	30.54	6.7 ± 0.1
			121	74.8 ± 1.0	15.1 ± 0.5	5.0 ± 0.8	33.11	6.9 ± 0.1

**Table 4:** Fatty acid methyl ester (FAME, biodiesel) detected from GC/Ms analysis.

Fatty Acid Methyl Ester	Common Name	Carbon Atoms: Double Bonds	% of Total	RT (min)
Dodecanoic acid, methyl ester	Methyl laurate	C12:0	8.497	22.17
Methyl tetradecanoate	Methyl myristate	C14:0	3.826	23.96
Pentadecanoic acid, methyl ester	Methyl pentadecanoate	C15:0	1.841	24.96
Hexadecanoic acid, methyl ester	Methyl palmitate	C16:0	20.156	26.24
9-Hexadecenoic acid, methyl ester, (Z)-	Methyl palmitoleate (Z-9)	C16:1	1.744	25.90
Methyl stearate	Methyl stearate	C18:0	8.289	29.42
7-Octadecenoic acid, methyl ester	Methyl octadecenoate	C18:1	21.763	29.11
9,12-Octadecadienoic acid, methyl ester	Methyl linoleate	C18:2	18.107	28.98
Hexadecanoic acid, 15-methyl-, methyl ester	Branched heptadecanoate methyl ester (iso)	br-C17:0	0.937	27.71
Hexadecanoic acid, 2-hydroxy-, methyl ester	Hydroxypalmitate methyl ester	OH-C16:0	4.645	38.14
Methyl 18-methylnonadecanoate	Branched C20 methyl ester	br-C20:0	0.878	31.74
2-Decenoic acid, methyl ester	Short-chain unsaturated methyl ester	C10:1	0.787	16.24
7-Nonenoic acid, methyl ester	Short-chain unsaturated methyl ester	C9:1	6.337	20.77
6-Heptenoic acid, methyl ester	Short-chain unsaturated methyl ester	C7:1	1.640	16.39
Dodecanoic acid, 12-(4-methylphenylsulfonyloxy)-, methyl ester	Modified dodecanoate methyl ester	—	0.560	30.59

**Table 5:** The properties of the produced biodiesel.

Property	Biodiesel Properties	Standard Biodiesel Properties	
		EN 14214 (B100)	ASTM D6751 (B100)
Density, ρ at 15 °C	873.8 kg m^−3^	860–900 kg m^−3^	No density limit
Kinematic viscosity, ν at 40 °C	4.11 mm^2^ s^−1^	3.5–5.0 mm^2^ s^−1^	1.9–6.0 mm^2^ s^−1^
Saponification number, SN	203.65 mg KOH g^−1^	ND	ND
Iodine value, IV	61.18 g I_2_/100 g	≤120 g I_2_/100 g	ND
Higher heating value, HHV	40.16 MJ kg^−1^	typical biodiesel ~37.5–41	ND
Cetane number, CN	60.3	≥51	≥45–47

**Table 6:** GC/Ms analysis of *Talaromyces atroroseus* QA2602 (PZ091940) pigments.

Compound	% of Total	RT (min.)
Propionic acid, 4-hydroxy-3-hexyl ester	7.926972909	12.353
2-Methylbutanoic anhydride	12.04358068	10.464
3-Methyl-2-butenoic acid, 4-hexadecyl ester	14.71142521	11.557
Butyric acid, 4-pentadecyl ester	4.776207303	16.644
1-Propoxypropan-2-yl 3-methylbutanoate	10.41224971	19.717
2,5-Dimethyl-7,7-diphenyl-3-aza-4,6-dioxabicyclo[3.2.0]hept-2-ene	1.537102473	23.305
8-Azabicyclo[3.2.1]octan-3-ol, 8-(2-hydroxy-2,2-diphenylethyl)-	48.61601885	26.772

**Table 7:** FTIR analysis of fungal chitosan and chitosan/pigment composite.

Functional Groups	Fungal Chitosan (cm^−1^)	Chitosan/Pigment Composite (cm^−1^)
O–H and N–H stretching; H-bonding	3451.71	3412.67
Aliphatic C–H (–CH_2_/–CH_3_) stretch	—	2931.60
CO_2_ (ambient)	—	2361.35
Amide I (C=O) ± π–π/H-bond effects	1630.55	1627.43
CH_2_ bending (scissoring)	—	1415.41
CH_3_ bending/amide III contrib.	1383.77	1380.86
Amide III (C–N) + CH	1323.83	1326.41
C–O stretching (pyranose)	—	1281.01
C–N stretching/skeletal	—	1261.01
C–O–C stretching (pyranose ring)	1029.31	986.11
β-1,4-glycosidic linkage vibration	—	886.14
C–H out-of-plane; possible aromatic contribution	697.95	668.14
Low-freq skeletal/ring deformation	—	589.25

## Data Availability

The sequencing data of the fungal strain *Talaromyces atroroseus* are strain QA2602 and they are deposited in the NCBI (http://www.ncbi.nlm.nih.gov) website under accession numbers; PZ091940.
